# Kaixin-San improves Aβ-induced synaptic plasticity inhibition by affecting the expression of regulation proteins associated with postsynaptic AMPAR expression

**DOI:** 10.3389/fphar.2023.1079400

**Published:** 2023-02-14

**Authors:** Bo Zhang, Meng-Lu Wang, Shu-Ming Huang, Yu Cui, Yan Li

**Affiliations:** ^1^ Institute of Chinese Medicine, Heilongjiang University of Chinese Medicine, Harbin, China; ^2^ Department of Veterinary Medicine, Institute of tropical agriculture and forestry, Hainan University, Haikou, China; ^3^ Jiangsu Key Laboratory of Integrated Traditional Chinese and Western Medicine for Prevention and Treatment of Senile Diseases, Medical College of Yangzhou University, Yangzhou, China

**Keywords:** Kaixin-San, amyloid-β protein, memory, long-term potentiation, AMPAR

## Abstract

**Objective:** To explore the mechanism underlying Kaixin-San (KXS) regulation of postsynaptic AMPA receptor (AMPAR) expression to mitigate toxic effects of the amyloid-β protein (Aβ).

**Methods:** An animal model was established *via* intracerebroventricular injection of Aβ1–42. The Morris water maze test was conducted to evaluate learning and memory, while electrophysiological recording was conducted to assess the hippocampal long-term potentiation (LTP). Western blotting was used to detect expression levels of the hippocampal postsynaptic AMPAR and its accessory proteins.

**Results:** The time spent to find the platform was significantly prolonged, the number of mice crossing the target site was significantly reduced, and the maintenance of LTP was inhibited in the Aβ group than in the control group. In the Aβ/KXS group, the time taken to find the platform was significantly shortened and the number of mice crossing the target site was significantly increased than in the Aβ group; furthermore, the inhibition of LTP induced by Aβ was reversed. The expression of GluR1, GluR2, ABP, GRIP1, NSF, and pGluR1–Ser845 was upregulated, while that of pGluR2–Ser880 and PKC δ was downregulated in the Aβ/KXS group.

**Conclusion:** The increased expression of ABP, GRIP1, NSF, and pGluR1–Ser845 and the decreased expression of pGluR2–Ser880 and PKC δ under the influence of KXS, followed by the upregulation of postsynaptic GluR1 and GluR2, alleviated the inhibition of LTP induced by Aβ. Ultimately, the memory function of model animals was improved by KXS. Our study provides novel insights into the mechanism underlying KXS mitigation of Aβ-induced synaptic plasticity inhibition and memory impairment by altering the levels of accessory proteins associated with AMPAR expression.

## 1 Introduction

Kaixin-San (KXS), used for the treatment of memory dysfunction in traditional Chinese medicine (TCM), consists of Ginseng Radix (*Panax ginseng* C. A. Mey.), Poria (*Poria cocos* (Schw.) Wolf), Radix Polygalae (*Polygala tenuifolia* Willd), and Acori Tatarinowii Rhizoma (*Acorus tatarinowii* Schott). Previous studies have shown that KXS can effectively improve the memory of patients with Alzheimer’s disease (AD) and mild cognitive impairment (Chen et al., 2016; Fang et al., 2017), and attenuate the memory dysfunction of model animals (Nishiyama et al., 1994; Guo et al., 2019; Xu et al., 2019). However, its mechanisms of action remain unclear.

Hippocampal long-term potentiation (LTP) is essential for memory formation (Bliss and Collingridge, 1993; Barnes, 2003). The basis for the LTP formation is alterations in the function of the α-amino-3-hydroxy-5-methyl-4-isoxazolepropionic acid (AMPA) receptor (AMPAR) on the postsynaptic membrane, which increases in sensitivity to presynaptic transmitter release (Hussain et al., 2014; Diering and Huganir, 2018). In hippocampal neurons, AMPARs comprise subunits (GluR1–GluR4). The GluR4 expression level is high in the early development of the hippocampus (Nuriya et al., 2005). In a mature hippocampus, GluR1/GluR2 and GluR2/GluR3 subunit combinations are the main forms of AMPAR assembly, with a small contribution of GluR1 homomers (Díaz-Alonso and Nicoll, 2021). GluR1 is rapidly recruited for synapses during LTP, while GluR2/GluR3 is recruited for synapses that gradually replace receptors that contain GluR1 at potentiated synapses (Shi et al., 2001). This subunit exchange occurring after LTP is considered to be an important part of memory consolidation (Diering and Huganir, 2018).

After reaching the surface of the postsynaptic membrane, AMPARs need to be fixed or anchored onto the synaptic membrane to exert their function. The following receptor accessory proteins mainly mediate this process: the glutamate receptor interacting protein (GRIP), AMPA receptor binding protein (ABP), and N-ethylmaleimide-sensitive factor (NSF). The combination of the AMPAR and ABP/GRIP can maintain the expression of AMPARs (Lu and Ziff, 2005), and the NSF can block AMPARs separated from the postsynaptic membrane, which is conducive for the maintenance of LTP (Hanley, 2007). Protein kinase A (PKA) could phosphorylate the GluR1–Ser845 site, and the phosphorylation of Ser845 promotes GluR1 targeting or retention on the cell surface, which is closely related to LTP maintenance (Oh et al., 2006). Moreover, phosphorylation by protein kinase C (PKC) can also dissociate AMPARs from the anchoring protein and initiate the endocytosis process. PKC phosphorylates Ser880 of GluR2 (McDonald et al., 2001), thereby weakening the affinity with the GRIP and reducing the enrichment of AMPARs on the postsynaptic membrane (Osten et al., 2000). The amyloid-β protein (Aβ), whose soluble oligomers have potent neurotoxicity and memory impairment properties, is a primary contributor to the pathogenesis of AD (Wilcox et al., 2011; Takahashi et al., 2021). We have previously shown that KXS increases postsynaptic GluR2 expression while improving Aβ-induced synaptic plasticity inhibition and memory impairment (Zhang et al., 2019), although the underlying mechanism remains to be elucidated.

The expression levels of the AMPAR on the postsynaptic membrane are influenced by anchoring, externalization, and endocytosis processes (Braithwaite et al., 2002; Patten and Ali, 2009; Park et al., 2016; Watson et al., 2017). To explore the mechanism underlying KXS-induced changes in AMPAR expression, in this study, we investigated the molecular expression of AMPAR-related proteins associated with the inhibitory effect of Aβ and the mitigating effect of KXS on the postsynaptic AMPAR, to elucidate the molecular mechanism underlying the beneficial effects of KXS on Aβ-induced memory impairment and provide experimental evidence to support the clinical application of KXS.

## 2 Materials and methods

### 2.1 Animals

Adult male Institute of Cancer Research (ICR) mice (25–30 g; 10 weeks old), provided by the Heilongjiang University of Chinese Medicine, were maintained at ambient temperature (24 ± 2°C) and humidity (55% ± 5%) on a 12-h dark/light cycle with unlimited access to food and water. All animal experiments were performed according to the Guide for the Care and Use of Laboratory Animals by the National Academy of Sciences and were approved by the Animal Experiment Ethics Committee of the Heilongjiang University of Chinese Medicine. During the experiments, all efforts were made to humanize the experiments for animals.

### 2.2 Kaixin-San preparation

KXS comprised Ginseng Radix, Poria, Radix Polygalae, and Acorus Tatarinowii Rhizoma mixed in a ratio of 3:3:2:2 by weight. KXS was obtained *via* extraction as described previously (Liu et al., 2014). Briefly, KXS was refluxed and extracted three times with a 10-fold quantity of 60% ethanol, each for 1.5 h. The extracts were dried under a vacuum and stored at −20°C for later use.

### 2.3 Lateral ventricle injection and drug administration

Mice were randomly divided into the control group, Aβ group, and Aβ/KXS group. Following anesthesia with an intraperitoneal injection of pentobarbital sodium (45 mg kg^-1^), the mice were fixed in a stereotaxic locator (1 mm posterior to the bregma and 1.75 mm lateral to the midline) and a microsyringe was vertically inserted into the brain to a depth of 1.8 mm, through which Aβ1–42 (1 μM and 5 μL; Sigma, A9810) was slowly injected into the unilateral ventricle for 5 min in both Aβ and Aβ/KXS groups. The control group mice were injected with an equal volume of normal saline at the same location in the brain. The mice in the Aβ/KXS group were administered KXS intragastrically (0.2 mL; 0.15 g kg^-1^) for 7 days (Zhang et al., 2019), while those in the remaining groups were administered an equal volume of normal saline once a day.

### 2.4 Behavioral experiments

The Morris water maze (MWM) test was used to evaluate the learning and memory function of all groups of mice (Bromley-Brits et al., 2011; Zhang et al., 2021). In the experiment, a black circular pool (for white mice) with a diameter of 150 cm and a depth of 50 cm filled with clear water (24 ± 2°C) was used. A hidden platform was placed 1.5 cm under the water surface at the center of the first quadrant of the pool. The experiment was divided into the following two parts: the positioning sailing training and spatial exploration experiment. Mice were gently placed into the water facing the pool wall at fixation points and tested once for 60 s in each quadrant (four quadrants in total). During the training session, whether or not the mouse found the platform within 60 s, it was allowed to stay on the platform for 10 s. The time the mice spent in finding the submerged platform was recorded each day.

After the training sessions, the spatial exploration experiment was conducted as the test phase. The platform was removed, and 2 h later, the mice were gently placed in the water at one point facing the pool wall in the fixed quadrant (quadrant II). The number of times the mice crossed the target site (the original platform position) within 60 s was counted. The movements of each mouse during training and test phases were recorded by a digital camera 10Moons SDK-2000 (10Moons, Huizhou, China) and were analyzed using the Morris water maze video analysis system V2.0 (Anhui Zhenghua, Huaibei, China).

### 2.5 Electrophysiological recording

Electrophysiological recording was performed as previously described (Zhang et al., 2019). Briefly, the stimulating electrode was positioned at the perforant path branch at 4.5 mm posterior to the bregma, 3.0 mm lateral to the midline, and 1.5–2.0 mm from the surface of the cortex. The recording electrode was inserted into the molecular layer of the hippocampal dentate gyrus (DG) area at 2.1 mm posterior to the bregma, 1.5 mm lateral to the midline, and at a depth of 1.75–2.25 mm from the surface of the cortex. A reference electrode was attached to the head skin.

We generated the input–output curve (I–O curve) by gradually increasing the intensity of the stimulation in the DG area before recording LTP, and the field excitatory postsynaptic potential (fEPSP) was evoked and recorded *via* electrical stimulation with a wave width of 0.6 ms and frequency of 0.067 Hz. The rising slope of the fEPSP in each mouse was obtained to evaluate the basic function of synaptic transmission. The electrical stimulation intensity that induced 30% of the maximum amplitude of the fEPSP was set as the stimulus intensity for the baseline fEPSP recording. After 15 min of stable baseline recording, high-frequency stimuli (HFS) (100 Hz; 100 trains; 1 s) were delivered to induce LTP. Then, fEPSPs were continuously monitored for 90 min. The fEPSPs were recorded using Clampex 10.2 software and analyzed using Clampfit 10.2 software (Molecular Devices Corporation, California, USA). The obtained data are presented as the percentage of the population spike (PS) amplitude of fEPSPs to the baseline and expressed as the mean ± standard error (SE).

### 2.6 Western blotting

Immediately following the electrophysiological experiment, mice were sacrificed by decapitation and the hippocampi were removed. The stimulated hippocampus was lysed on ice for 30 min and the supernatant was centrifuged (12,000 × *g*; 30 min; 4°C) to obtain the total protein. A standard protein concentration curve was established and the protein concentration in the mouse hippocampus was determined using the BCA Protein Assay Kit (Beyotime, P0012S). The loading buffer (4×) was added to the protein according to the measured protein concentration and boiled for 10 min and centrifuged (12,000 × g; 5 min; 4°C). Then, 20 μg of protein samples were loaded per well, and the proteins were separated by SDS-PAGE (8% separation gel and 5% concentrated gel) using electrophoresis parameters (upper layer gel, 70 V for 30 min; lower layer gel, 110 V for 1 h). The proteins were transferred onto PVDF membranes *via* wet transfer using 100 V constant pressure for 2 h. After 1 h of incubation with the Western blocking solution at room temperature, the membranes were incubated with one of the primary antibodies (anti-GluR1 [1:2,000, ab109450; Abcam]; anti-GluR2 [1:2,000, ab206293; Abcam]; anti-phospho-GluR1 [Ser845, ab222761; Abcam]; anti-phospho-GluR2 [Ser880, bs-5359R; Bioss]; anti-GluR2/3 [1:500, AF5458; Affinity]; anti-NSF [1:500, DF4611; Affinity]; anti-GRIP1 [1:500, DF2500; Affinity]; anti-ABP [1:500, bs-2410R; Bioss]; anti-PKA alpha + beta [1:500, bs-0520R; Bioss]; anti-beta-actin [1:5,000, bs-0061R; Bioss]; and anti-GAPDH [1:3,000, bs-2188R; Bioss]) at 4 °C overnight. After incubation with the peroxidase-conjugated secondary antibody (goat anti-rabbit IgG [1:7,500, ZSGB-BIO, ZB-2301]) at room temperature for 1 h, the electrochemiluminescence reagent (Meilunbio, Dalian, China) was applied to the membranes for signal detection. Finally, immunoblots were exposed to the gel imaging and analysis system (Tanon 5200, China).

### 2.7 Statistical analysis

A statistical analysis was conducted using SPSS 21.0 software; all data are expressed as the mean ± standard deviation (SD). Comparisons of all data among the groups were performed using the analysis of variance (ANOVA), while the comparison between two groups was carried out using a two-sample *t*-test. The statistical significance was set at *p* < 0.05.

## 3 Results

### 3.1 Kaixin-San improving learning and memory in mice

**FIGURE 1:**
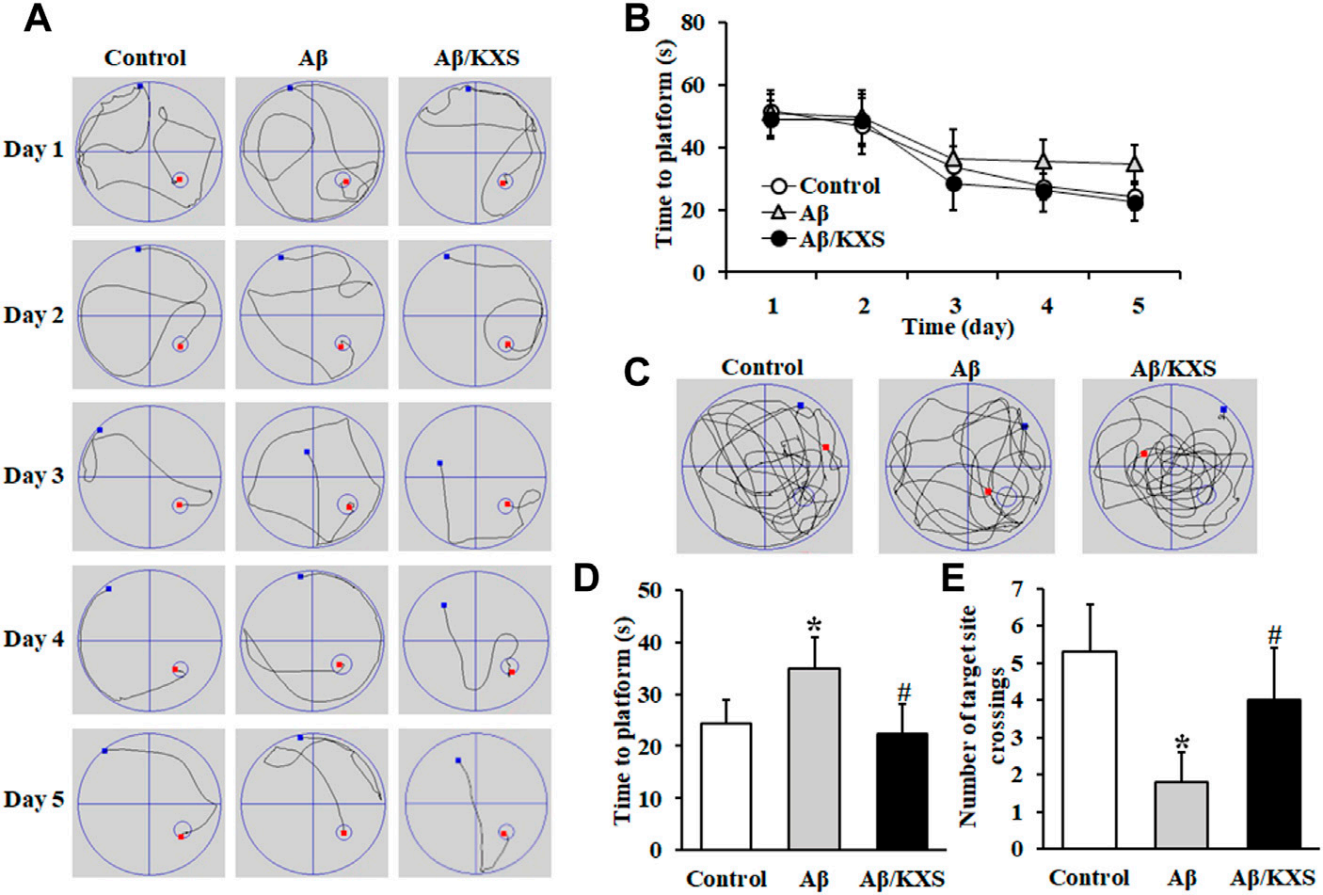
Effect of KXS on learning and memory of model mice. **(A)** Representative swimming traces of a control mouse (*Left*), an Aβ mouse (*Middle*), and an Aβ/KXS mouse (*Right*) during the training phase. **(B)** Time taken to find the hidden platform during five consecutive days of the positioning sailing training. There were significant differences in the time taken to find the platform in all groups on days 4 and 5. **(C)** Representative swimming traces of a control mouse (*Left*), an Aβ mouse (*Middle*), and an Aβ/KXS mouse (*Right*) during the test phase. **(D)** Statistical comparison of the time taken to find the platform on day 5. **(E)** Number of mice crossing the target site; n = 10 per group. Each column with a bar represents the mean ± SD. * *p* < 0.05, compared with the control group; #*p* < 0.05 compared with the Aβ group. Aβ, amyloid-β; KXS, Kaixin-San.

During the training phase, the time for the mice in the three groups to find the platform submerged in the water maze progressively decreased with the training time (Figures 1A,B). However, the magnitude of the decline was different in the three groups. The Aβ mice required a significantly longer duration to find the platform than the Aβ/KXS on days 4 and 5 (Figure 1B). The test results on day 5 are shown in Figures 1C–E. During the test phase, the control and Aβ/KXS mice persistently swam to and across the former platform location, whereas the Aβ mice did not. Compared with that in the control group, the time taken to find the platform in the Aβ group was significantly prolonged (*p* < 0.05), and the number of times the mice crossed the target site was significantly reduced (*p* < 0.05), indicating that Aβ impaired both learning and memory. Conversely, the mice in the Aβ/KXS group spent a shorter time in finding the platform (*p* < 0.05) than those in the Aβ group; furthermore, the number of mice crossing the target site was significantly increased in the former group (*p* < 0.05). These results suggest that KXS improved Aβ-induced memory impairment.

### 3.2 Kaixin-San reversing Aβ-induced long-term potentiation inhibition by upregulating the expression of postsynaptic GluR1 and GluR2

**FIGURE 2:**
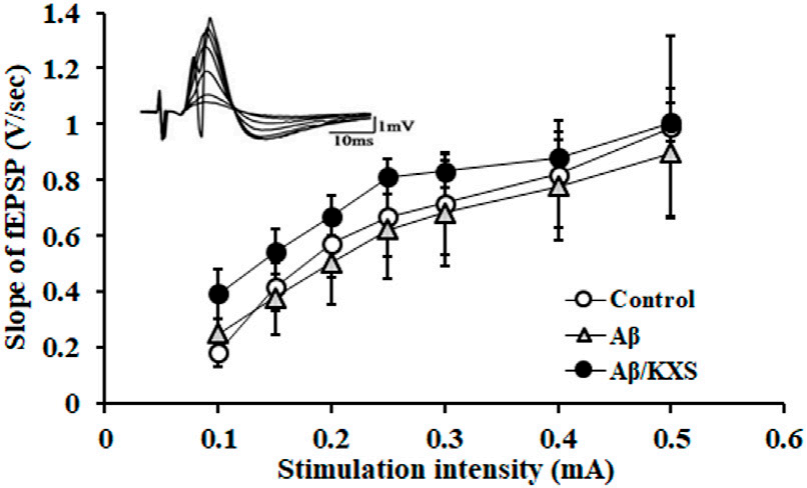
I–O curve recording in the experimental groups. There were no significant differences among these three groups. The waveforms of fEPSPs at different stimulation intensities are shown at the top left upper corner. Each value represents the mean ± SE.

We first tested the basal synaptic transmission by recording the I–O curve. The slope of the fEPSP was increased with a progressive increase of the electrical stimulation intensity, whereas there were no significant differences between these three groups (Figure 2). This indicated that the basal synaptic transmission of the mice in the experimental groups did not change.

**FIGURE 3:**
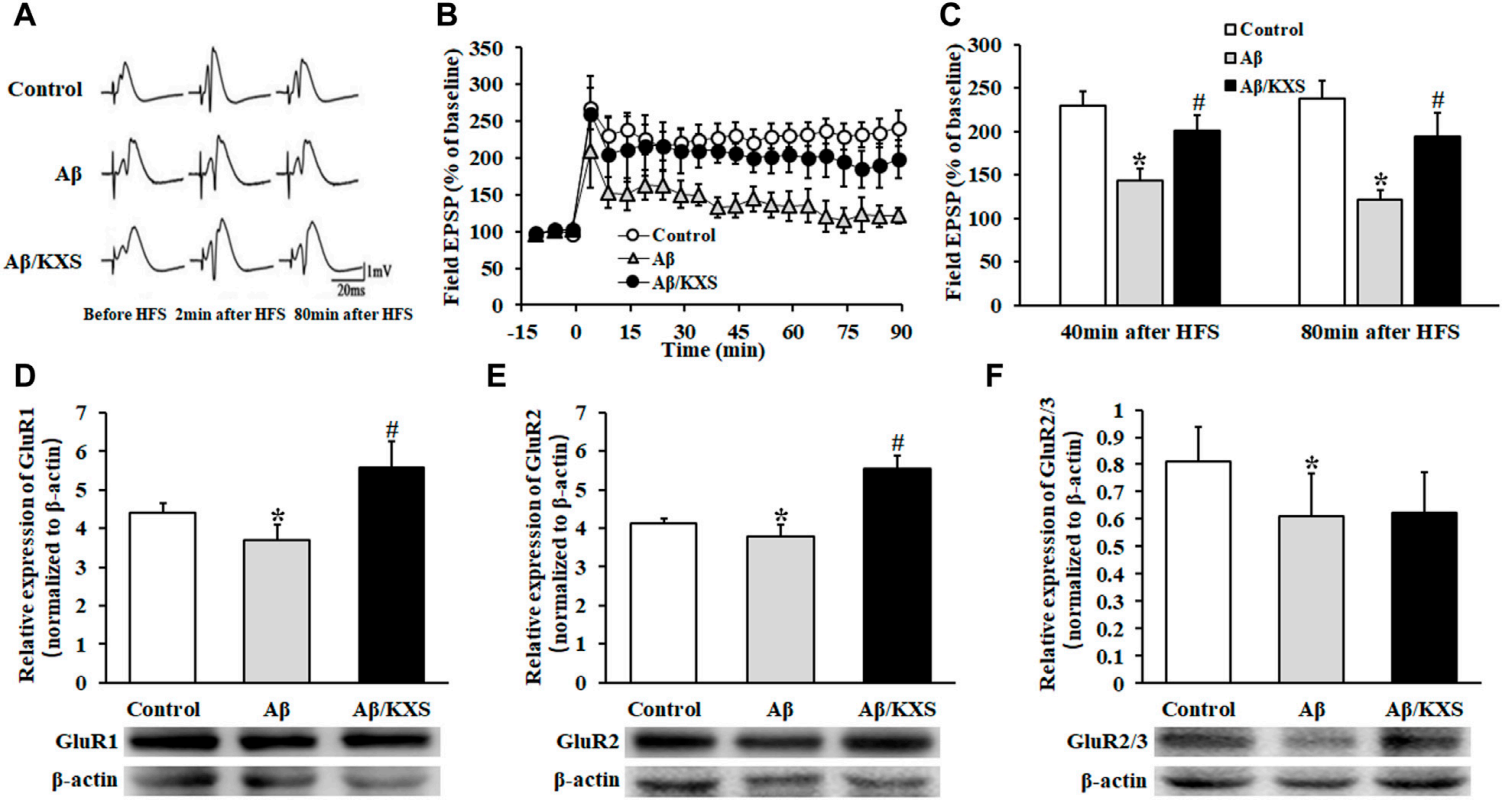
Effect of KXS on LTP and AMPAR expression. **(A)** fEPSP waveforms in each group at different stages. **(B)** Alterations in the fEPSP PS amplitude in each group at different time points. At 80 min post HFS, the PS amplitude in the Aβ/KXS group significantly increased compared with that in the Aβ group. Each value represents the mean ± SE. **(C)** Statistical differences of mice in three groups at different time points. **(D–F)** Statistical analysis of the GluR1, GluR2, and GluR2/3 expression performed following Western blotting. The pictures below the graph indicate the protein exposure; *n* = 6 per group. Each column with a bar represents the mean ± SD. * *p* < 0.05 versus the control group; #*p* < 0.05 versus the Aβ group.

The results of LTP recording are shown in Figures 3A–C; 80 min following HFS, the average population spike amplitude of the fEPSP (121.08% ± 12.02%; n = 6) was significantly weakened in the Aβ group as compared to that in the control group (237.30% ± 21.49%; n = 20; *p* < 0.05), and it was significantly enhanced in the Aβ/KXS group (193.93% ± 28.01%; n = 7; *p* < 0.05) compared to that in the Aβ group, suggesting that KXS improved Aβ-induced LTP inhibition. These results indicate that KXS can improve Aβ-induced memory impairment by reversing Aβ-induced LTP inhibition. Therefore, to elucidate the underlying mechanism, we assessed the expression of postsynaptic GluR1, GluR2, and GluR2/3.

As shown in Figures 3D–F, the expression of GluR1 and GluR2 in the Aβ group decreased compared with that in the control group (*p* < 0.05). However, a significantly increased expression of GluR1 and GluR2 was observed in the Aβ/KXS group (*p* < 0.05). KXS slightly increased the GluR2/3 expression in the Aβ/KXS group compared with that in the Aβ group, although not significantly, whereas Aβ decreased the expression of GluR2/3 in the Aβ group compared with that in the control group (*p* < 0.05). This suggests that KXS ameliorated Aβ-induced LTP inhibition by increasing the expression of GluR1 and GluR2. Further investigations are warranted to elucidate the underlying mechanism.

### 3.3 Kaixin-San increasing the expression of postsynaptic ABP, GRIP1, NSF, and pGluR1–Ser845 and decreasing that of pGluR2–Ser880 and PKC δ

To explore the reason for the change of AMPAR expression, we detected the expression of proteins, including ABP, GRIP, NSF, pGluR1–Ser845, PKA, pGluR2–Ser880, and PKC, which were closely related to the anchoring, externalization, and endocytosis of AMPARs.

**FIGURE 4:**
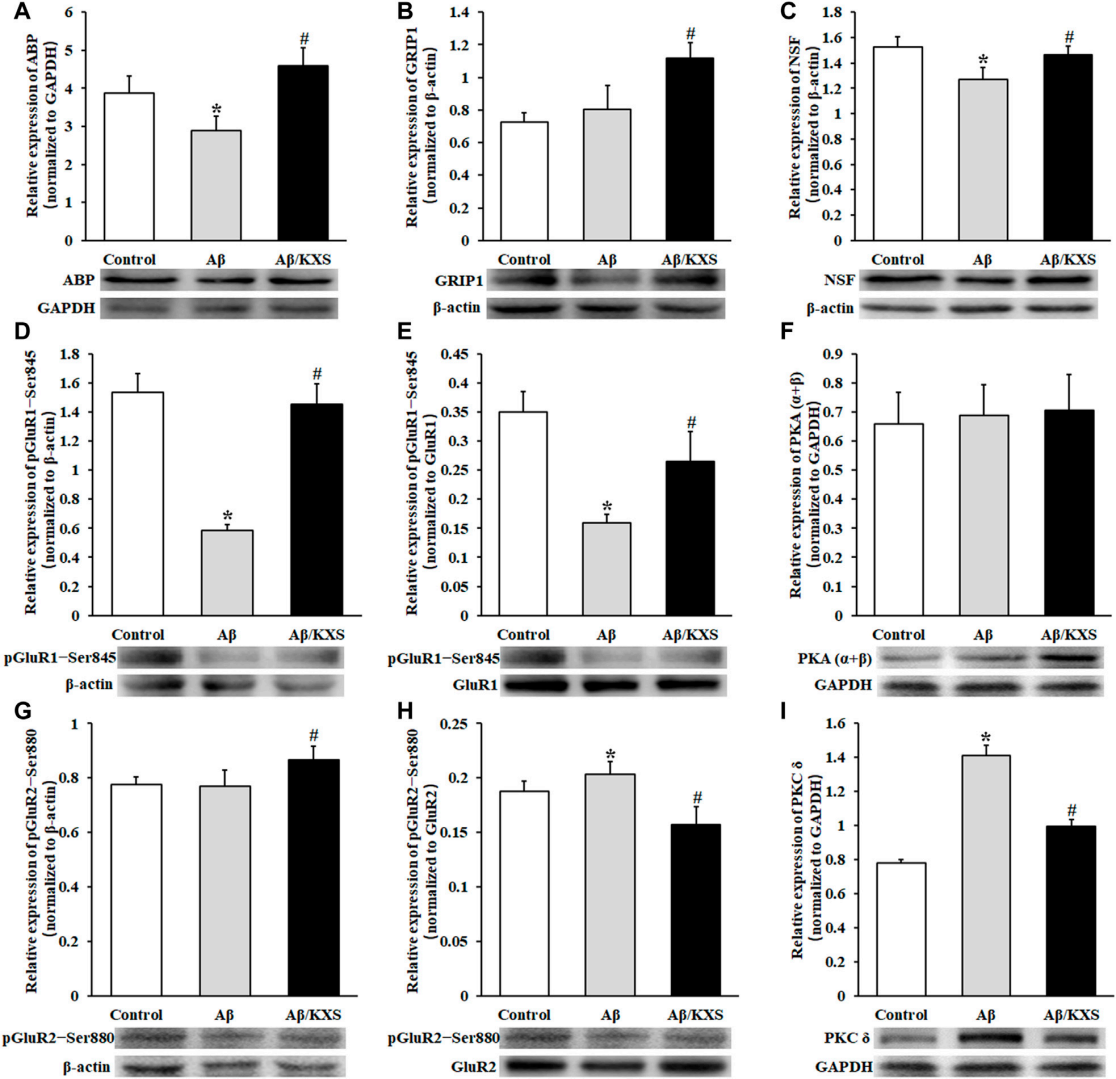
Effect of KXS on the levels of AMPAR subunits related to the protein expression. The statistical analysis of ABP **(A)**, GRIP1 **(B)**, NSF **(C)**, pGluR1–Ser845 **(D)**, pGluR1–Ser845/GluR1 **(E)**, PKA (α+β) **(F)**, pGluR2–Ser880 **(G)**, pGluR2–Ser880/GluR2 **(H)**, and PKC δ **(I)** expression was determined using Western blotting. The pictures below the graph indicate the protein exposure; n = 6 per group. Each column with a bar represents the mean ± SD. * *p* < 0.05 versus the control group; #*p* < 0.05 versus the Aβ group. During the experiment, GluR1 and pGluR1–Ser845, as well as GluR2 and pGluR2–Ser880, were run simultaneously on the same electrophoretic gel; hence, the same loading control is used in Figures 3D and 4D and in Figures 3E and 4G. The molecular weights of PKA (α + β) and PKC δ are 40 kD and 77 kD, respectively; therefore, they may be separated on one gel and transferred to the same PVDF membrane. The same loading control is used in Figures 4F,I.

Aβ reduced the expression of ABP and NSF, while KXS increased the expression of ABP and NSF (Figures 4A,C), suggesting that KXS can restore the expression of ABP and NSF inhibited by Aβ. Figure 4B shows that KXS increased the expression of GRIP1, while no significant difference in the GRIP1 expression between the Aβ and control groups was observed. These results suggest that KXS reversed the inhibition of Aβ on the ABP and NSF expression and upregulated the GRIP1 expression, thereby increasing postsynaptic AMPAR levels.

Figure 4D shows that the level of phosphorylation of GluR1 at Ser845 (pGluR1–Ser845) was decreased in the Aβ group (*p* < 0.05) but increased in the Aβ/KXS group (*p* < 0.05). Additionally, the relative ratio of pGluR1–Ser845 to the total GluR1 was calculated. Figure 4E shows that the pGluR1–Ser845/GluR1 ratio was increased in the Aβ/KXS group (*p* < 0.05) but decreased in the Aβ group when compared with that in the control group (*p* < 0.05). These results demonstrate that the level of pGluR1–Ser845 was increased in the Aβ/KXS group but decreased in the Aβ group. In an attempt to explain the increase in phosphorylation levels of GluR1 at the Ser845 site, we assessed the level of PKA. Our results showed no statistical difference among the groups in terms of PKA (α + β) expression (Figure 4F). This suggests that KXS might have increased AMPAR expression through the increased pGluR1–Ser845 expression, which was not achieved *via* PKA.

No significant change in the level of phosphorylation of GluR2 at Ser880 (pGluR2–Ser880) was observed in the Aβ group, while it was increased in the Aβ/KXS group (*p* < 0.05; Figure 4G). Furthermore, the Aβ group showed a higher ratio of pGluR2–Ser880 to GluR2 than the control group, while a lower ratio was shown in the Aβ/KXS group than the Aβ group (*p* < 0.05; Figure 4H), indicating that KXS decreased the level of pGluR2–Ser880, which was increased by Aβ. These results suggest that the increase in the GluR2 expression by KXS was correlated with the decrease in the pGluR2–Ser880 expression. To confirm the mechanism underlying the change in the GluR2–Ser880 phosphorylation expression, the level of PKC δ was examined. Figure 4I shows that the PKC δ expression was increased in the Aβ group but decreased in the Aβ/KXS group, suggesting that KXS can increase AMPAR expression levels by reducing the PKC δ expression and decreasing the levels of GluR2 phosphorylation at the Ser880 site.

## 4 Discussion

Aβ exhibits neurotoxic properties and impairs memory and cognitive function (Reiss et al., 2018). KXS is a TCM used to “cure forgetfulness” (Chen et al., 2016). To explore the effect of KXS on memory impairment induced by Aβ, an animal model was established *via* an Aβ intracerebral injection. Behavioral tests confirmed that KXS can improve Aβ-induced memory impairment, which is consistent with our previous findings (Zhang et al., 2019).

Previous studies have demonstrated that KXS can improve the memory of animal models of memory dysfunction through multiple pathways (Fu et al., 2019; Luo et al., 2020). It was reported that KXS enhanced the hippocampal LTP in normal rats (Smriga et al., 1995). However, data on the effect of KXS on synaptic plasticity, which is the electrophysiological basis for learning and memory, remain limited (Zhang et al., 1994; Smriga et al., 1995). Our previous findings confirmed that KXS improves synaptic plasticity in animal models and the involvement of postsynaptic GluR2 using immunohistochemistry (Zhang et al., 2019). This provides the basic idea for the present study, which is anchored to the related mechanism of the postsynaptic membrane. Since the postsynaptic AMPAR is the key to the induction and maintenance of LTP, we focused on the subunits of AMPARs and their accessory proteins. The increased GluR1 expression contributes to increased postsynaptic AMPAR sensitivity, thereby maintaining LTP (Andrásfalvy et al., 2003; Diering and Huganir, 2018; Jiang et al., 2021), while GluR2 and GluR2/3 are involved in the formation of LTP (Diering and Huganir, 2018). As the postsynaptic AMPAR is the key to the LTP formation (Biou et al., 2008; Díaz-Alonso and Nicoll, 2021), we assessed the expression of GluR1, GluR2, and GluR2/3 in this study and found that Aβ inhibited their expression, which is consistent with the results of previous studies (Miñano-Molina et al., 2011; Du et al., 2020). Moreover, KXS upregulated the expression of GluR1 and GluR2, but it had no effect on the expression of GluR2/3. Although GluR2 can block the calcium influx, our results showed that KXS increased the expression of GluR2, possibly due to LTP, which is influenced by various factors (Baltaci et al., 2019). Further investigations are warranted to elucidate the underlying mechanism.

Alternation in postsynaptic AMPAR expression is regulated by anchoring, externalization, and endocytosis proteins. Anchoring proteins facilitate the postsynaptic membrane surface AMPAR aggregation and maintenance of quantity (Dong et al., 1997), which is necessary for memory formation (Tan et al., 2020). Our results showed that KXS increased the expression of major anchoring proteins involved in postsynaptic AMPAR expression, including ABP, GRIP1, and NSF.

AMPAR subunit phosphorylation is an important step in AMPAR externalization to the postsynaptic membrane. Phosphorylation levels of GluR1 at Ser845 facilitate AMPAR externalization and enhance LTP (Esteban et al., 2003; Makino et al., 2011). KXS could increase pGluR1–Ser845 levels, which are regulated by PKA (Esteban et al., 2003); however, we observed no significant changes in PKA expression, which suggests other regulatory mechanisms for pGluR1–Ser845 (Catalano et al., 2008). During the induction of the hippocampal LTP, the Ca^2+^ influx activates calcium/calmodulin-dependent protein kinase II (CaMKII), which directly phosphorylates GluR1 at Ser831, increases the GluR1 channel conductance, and promotes GluR1 targeting PSD and the induction of hippocampal LTP (Barria et al., 1997). However, it was reported that the phosphorylation of GluR1 at the Ser831 site by CaMKII is not required for receptor binding or LTP (Esteban et al., 2003). It is complicated to explain the underlying mechanisms, which needs further investigation.

**FIGURE 5:**
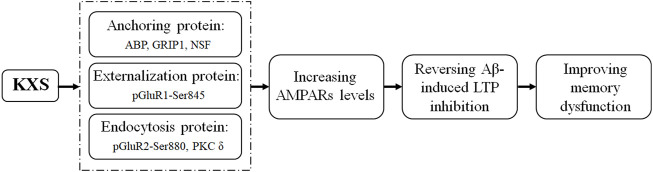
Illustration of the mechanism underlying KXS attenuation of memory impairment induced by Aβ.

Furthermore, KXS reduced the phosphorylation level of GluR2 at the Ser880 site, which blocked the binding of the receptors to the anchoring protein (Osten et al., 2000; Park et al., 2009) and promoted GluR2 endocytosis. The phosphorylation of GluR2 at Ser880 is PKC dependent (Chung et al., 2000); our findings showed that KXS reduced the expression of PKC δ, which was increased in the Aβ group, consistent with previous findings that increased the PKC δ level involved in memory impairment (Mai et al., 2018). In this study, we found that KXS increased the expression of ABP, GRIP1, NSF, and pGluR1–Ser845, suggesting an increase in the AMPAR moving out to the surface of the postsynaptic membrane. Simultaneously, the expression of PKC δ and pGluR2–Ser880 was reduced, suggesting a decrease in the endocytosis of AMPARs. These results suggest that KXS increased the number of postsynaptic GluR1 and GluR2 by regulating the expression of AMPAR-related anchoring, externalization, and endocytosis proteins, thereby enhancing the sensitivity of the AMPAR to neurotransmitters released from the presynaptic membrane. Therefore, the Aβ-induced memory impairment in mice was attenuated by KXS (Figure 5).

Calcium is involved in many biochemical and physiological processes in mammals, and it is also an important factor in the induction and maintenance of LTP. HFS that induces LTP depolarizes the postsynaptic membrane, leading to an increase in the postsynaptic Ca^2+^ concentration, subsequently activating downstream biochemical processes (Malenka, 1991), including CaMKII (Herring and Nicoll, 2016) and PKC. The activity of these proteins promotes more AMPARs incorporated into the postsynaptic membrane during the maintenance of LTP (Sheng and Kim, 2002). However, calcium is also involved in the initiation of AMPAR endocytosis (Beattie et al., 2000). In the present study, whether KXS regulates postsynaptic receptor accessory proteins through calcium signaling is unclear, which needs to be elucidated by more studies in the future.

In addition, Aβ mice present short-term but not long-term synaptic plasticity. HFS can cause a large number of Ca^2+^ to enter presynaptic terminals. Temporary and high levels of accumulation of free Ca^2+^ activated calcium-sensitive enzymes, such as CaMKII, thereby promoting the mobilization of synaptic vesicles, resulting in massive release of neurotransmitters, and leading to the potentiation of synaptic transmission. However, the amplitude of the fEPSP progressively decreased with prolonged time in Aβ mice, and the maintenance of LTP was inhibited. This may be related to the decrease of ABP, NSF, and pGluR1–Ser845, and the increase of pGluR2–Ser880 and PKC δ, resulting in the decrease of the GluR1, GluR2, and GluR2/3 expression in the postsynaptic membrane.

TCM prescriptions often involve multiple pathways and multiple targets; hence, KXS may act on presynaptic and postsynaptic membranes, in conjunction with other mechanisms. The production and trafficking of AMPARs during LTP involved complex dynamic processes such as the modification of AMPAR subunits and the effects of AMPAR auxiliary subunits (Diering and Huganir, 2018; Park, 2018). Moreover, the role of synaptic adhesion molecules in AMPAR anchoring has gained considerable attention (Bhouri et al., 2018). Herein, we focused on the possible regulatory mechanisms of the postsynaptic AMPAR; the effect of KXS on presynaptic membranes and other mechanisms remains to be investigated. Interestingly, the hippocampal LTP could be enhanced by KXS in normal rats (Smriga et al., 1995). This suggests that KXS directly acts on AMPAR accessory proteins without the presence of Aβ. Considered with our present findings, this raised a new idea that KXS may be used in most of the diseases with reduced synaptic plasticity, which is highly interesting and requires further investigation.

## 5 Conclusion

By upregulating the expression of ABP, NSF, GRIP1, and pGluR1–Ser845 and decreasing the expression of pGluR2–Ser880 and PKC δ, KXS increased the level of postsynaptic AMPARs to reverse Aβ-induced LTP inhibition and, consequently, the memory dysfunction in our animal model. These results provide novel insights into the molecular mechanisms underlying the effects of KXS in improving Aβ-induced memory impairment and will help facilitate the clinical use of KXS against Aβ-associated memory loss or Aβ-induced neurotoxicity such as that in AD.

## Data Availability

The original contributions presented in the study are included in the article/Supplementary Material; further inquiries can be directed to the corresponding authors.

## References

[B1] Andrásfalvy B. K. Smith M. A. Borchardt T. Sprengel R. Magee J. C. (2003). Impaired regulation of synaptic strength in hippocampal neurons from GluR1-deficient mice. J. Physiol. 552, 35–45. Epub 2003/07/25. 10.1113/jphysiol.2003.045575 12878757 PMC2343312

[B2] Baltaci S. B. Mogulkoc R. Baltaci A. K. (2019). Molecular mechanisms of early and late LTP. Neurochem. Res. 44 (2), 281–296. Epub 2018/12/14. 10.1007/s11064-018-2695-4 30523578

[B3] Barnes C. A. (2003). Long-term potentiation and the ageing brain. Philosophical Trans. R. Soc. Lond. Ser. B, Biol. Sci. 358 (1432), 765–772. Epub 2003/05/13. 10.1098/rstb.2002.1244 PMC1693160 12740124

[B4] Barria A. Derkach V. Soderling T. (1997). Identification of the Ca2+/calmodulin-dependent protein kinase Ⅱ regulatory phosphorylation site in the alpha-amino-3-hydroxyl-5-methyl-4-isoxazole-propionate-type glutamate receptor. J. Biol. Chem. 272 (52), 32727–32730. Epub 1998/01/31. 10.1074/jbc.272.52.32727 9407043

[B5] Beattie E. C. Carroll R. C. Yu X. Morishita W. Yasuda H. von Zastrow M. (2000). Regulation of AMPA receptor endocytosis by a signaling mechanism shared with LTD. Nat. Neurosci. 3 (12), 1291–1300. Epub 2000/12/02. 10.1038/81823 11100150

[B6] Bhouri M. Morishita W. Temkin P. Goswami D. Kawabe H. Brose N. (2018). Deletion of LRRTM1 and LRRTM2 in adult mice impairs basal AMPA receptor transmission and LTP in hippocampal CA1 pyramidal neurons. Proc. Natl. Acad. Sci. U. S. A. 115 (23), E5382–E5389. Epub 2018/05/23. 10.1073/pnas.1803280115 29784826 PMC6003336

[B7] Biou V. Bhattacharyya S. Malenka R. C. (2008). Endocytosis and recycling of AMPA receptors lacking GluR2/3. Proc. Natl. Acad. Sci. U. S. A. 105 (3), 1038–1043. Epub 2008/01/16. 10.1073/pnas.0711412105 18195348 PMC2242698

[B8] Bliss T. V. Collingridge G. L. (1993). A synaptic model of memory: Long-term potentiation in the Hippocampus. Nature 361 (6407), 31–39. Epub 1993/01/07. 10.1038/361031a0 8421494

[B9] Braithwaite S. P. Xia H. Malenka R. C. (2002). Differential roles for NSF and GRIP/ABP in AMPA receptor cycling. Proc. Natl. Acad. Sci. U. S. A. 99 (10), 7096–7101. Epub 2002/05/16. 10.1073/pnas.102156099 12011465 PMC124534

[B10] Bromley-Brits K. Deng Y. Song W. (2011). Morris water maze test for learning and memory deficits in Alzheimer's disease model mice. J. Vis. Exp. JoVE (53), 2920. Epub 2011/08/03. 10.3791/2920 21808223 PMC3347885

[B11] Catalano M. Trettel F. Cipriani R. Lauro C. Sobrero F. Eusebi F. (2008). Chemokine CXCL8 modulates GluR1 phosphorylation. J. Neuroimmunol. 198 (1-2), 75–81. Epub 2008/05/30. 10.1016/j.jneuroim.2008.04.017 18508130

[B12] Chen Y. C. Bai Y. Wang Y. Q. Wang H. Yu J. L. Luo K. X. (2016). Clinical observation of modifed Kaixin yizhi decoction according to syndrome differentiation on treating mild cognitive impairment. China J. Traditional Chin. Med. Pharm. 31 (12), 5370–5372. Epub 2016/12/01.

[B13] Chung H. J. Xia J. Scannevin R. H. Zhang X. Huganir R. L. (2000). Phosphorylation of the AMPA receptor subunit GluR2 differentially regulates its interaction with PDZ domain-containing proteins. J. Neurosci. 20 (19), 7258–7267. Epub 2000/09/29. 10.1523/jneurosci.20-19-07258.2000 11007883 PMC6772789

[B14] Díaz-Alonso J. Nicoll R. A. (2021). AMPA receptor trafficking and LTP: Carboxy-termini, amino-termini and TARPs. Neuropharmacology 197, 108710. Epub 2021/07/17. 10.1016/j.neuropharm.2021.108710 34271016 PMC9122021

[B15] Diering G. H. Huganir R. L. (2018). The AMPA receptor code of synaptic plasticity. Neuron 100 (2), 314–329. Epub 2018/10/26. 10.1016/j.neuron.2018.10.018 30359599 PMC6214363

[B16] Dong H. O'Brien R. J. Fung E. T. Lanahan A. A. Worley P. F. Huganir R. L. (1997). GRIP: A synaptic PDZ domain-containing protein that interacts with AMPA receptors. Nature 386 (6622), 279–284. Epub 1997/03/20. 10.1038/386279a0 9069286

[B17] Du Y. Fu M. Huang Z. Tian X. Li J. Pang Y. (2020). TRPV1 activation alleviates cognitive and synaptic plasticity impairments through inhibiting AMPAR endocytosis in APP23/PS45 mouse model of Alzheimer's disease. Aging Cell 19 (3), e13113. Epub 2020/02/16. 10.1111/acel.13113 32061032 PMC7059138

[B18] Esteban J. A. Shi S. H. Wilson C. Nuriya M. Huganir R. L. Malinow R. (2003). PKA phosphorylation of AMPA receptor subunits controls synaptic trafficking underlying plasticity. Nat. Neurosci. 6 (2), 136–143. Epub 2003/01/22. 10.1038/nn997 12536214

[B19] Fang Y. Wang L. J. Guo Z. W. Chen X. L. Chen Y. C. (2017). Clinical observation on 30 cases of Alzheimer's disease treated with Kaixin Yizhi decoction. Zhejiang J. Traditional Chin. Med. 52 (12), 882–883. 10.13633/j.cnki.zjtcm.2017.12.014

[B20] Fu H. Xu Z. Zhang X. L. Zheng G. Q. (2019). Kaixinsan, a well-known Chinese herbal prescription, for Alzheimer's disease and depression: A preclinical systematic review. Front. Neurosci. 13, 1421. Epub 2020/02/06. 10.3389/fnins.2019.01421 32009890 PMC6971218

[B21] Guo S. Wang J. Xu H. Rong W. Gao C. Yuan Z. (2019). Classic prescription, Kai-Xin-San, ameliorates Alzheimer's disease as an effective multitarget treatment: From neurotransmitter to protein signaling pathway. Oxidative Med. Cell. Longev. 2019, 9096409. Epub 2019/07/30. 10.1155/2019/9096409 PMC6636599 31354916

[B22] Hanley J. G. (2007). NSF binds calcium to regulate its interaction with AMPA receptor subunit GluR2. J. Neurochem. 101 (6), 1644–1650. Epub 2007/02/17. 10.1111/j.1471-4159.2007.04455.x 17302911 PMC1976250

[B23] Herring B. E. Nicoll R. A. (2016). Long-term potentiation: From camkⅡ to AMPA receptor trafficking. Annu. Rev. physiology 78, 351–365. Epub 2016/02/11. 10.1146/annurev-physiol-021014-071753 26863325

[B24] Hussain N. K. Diering G. H. Sole J. Anggono V. Huganir R. L. (2014). Sorting nexin 27 regulates basal and activity-dependent trafficking of AMPARs. Proc. Natl. Acad. Sci. U. S. A. 111 (32), 11840–11845. Epub 2014/07/30. 10.1073/pnas.1412415111 25071192 PMC4136608

[B25] Jiang C. H. Wei M. Zhang C. Shi Y. S. (2021). The amino-terminal domain of GluA1 mediates LTP maintenance via interaction with neuroplastin-65. Proc. Natl. Acad. Sci. U. S. A. 118 (9), e2019194118. Epub 2021/02/26. 10.1073/pnas.2019194118 33627404 PMC7936340

[B26] Liu X. W. Liu S. Huang S. M. (2014). Plasma pharmacochemistry study of effective extracivet from Kai-Xinsan on Alzhemer's disease. Chin. J. Exp. Traditional Med. Formulae 20 (6), 179–183. Epub 2014/03/20. 10.11653/syfj2014060179

[B27] Lu W. Ziff E. B. (2005). PICK1 interacts with ABP/GRIP to regulate AMPA receptor trafficking. Neuron 47 (3), 407–421. Epub 2005/08/02. 10.1016/j.neuron.2005.07.006 16055064

[B28] Luo Y. Li D. Liao Y. Cai C. Wu Q. Ke H. (2020). Systems Pharmacology approach to investigate the mechanism of Kai-Xin-San in Alzheimer's disease. Front. Pharmacol. 11, 381. Epub 2020/04/23. 10.3389/fphar.2020.00381 32317964 PMC7147119

[B29] Mai H. N. Sharma N. Shin E. J. Nguyen B. T. Nguyen P. T. Jeong J. H. (2018). Exposure to far-infrared ray attenuates methamphetamine-induced impairment in recognition memory through inhibition of protein kinase C δ in male mice: Comparison with the antipsychotic clozapine. J. Neurosci. Res. 96 (7), 1294–1310. Epub 2018/02/25. 10.1002/jnr.24228 29476655

[B30] Makino Y. Johnson R. C. Yu Y. Takamiya K. Huganir R. L. (2011). Enhanced synaptic plasticity in mice with phosphomimetic mutation of the GluA1 AMPA receptor. Proc. Natl. Acad. Sci. U. S. A. 108 (20), 8450–8455. Epub 2011/05/04. 10.1073/pnas.1105261108 21536866 PMC3100939

[B31] Malenka R. C. (1991). The role of postsynaptic calcium in the induction of long-term potentiation. Mol. Neurobiol. 5 (2-4), 289–295. Epub 1991/01/01. 10.1007/bf02935552 1668390

[B32] McDonald B. J. Chung H. J. Huganir R. L. (2001). Identification of protein kinase C phosphorylation sites within the AMPA receptor GluR2 subunit. Neuropharmacology 41 (6), 672–679. Epub 2001/10/20. 10.1016/s0028-3908(01)00129-0 11640921

[B33] Miñano-Molina A. J. España J. Martín E. Barneda-Zahonero B. Fadó R. Solé M. (2011). Soluble oligomers of amyloid-β peptide disrupt membrane trafficking of α-amino-3-hydroxy-5-methylisoxazole-4-propionic acid receptor contributing to early synapse dysfunction. J. Biol. Chem. 286 (31), 27311–27321. Epub 2011/06/15. 10.1074/jbc.M111.227504 21665950 PMC3149325

[B34] Nishiyama N. Zhou Y. Takashina K. Saito H. (1994). Effects of DX-9386, a traditional Chinese preparation, on passive and active avoidance performances in mice. Biol. Pharm. Bull. 17 (11), 1472–1476. Epub 1994/11/01. 10.1248/bpb.17.1472 7703966

[B35] Nuriya M. Oh S. Huganir R. L. (2005). Phosphorylation-dependent interactions of alpha-actinin-1/IQGAP1 with the AMPA receptor subunit GluR4. J. Neurochem. 95 (2), 544–552. Epub 2005/09/30. 10.1111/j.1471-4159.2005.03410.x 16190873

[B36] Oh M. C. Derkach V. A. Guire E. S. Soderling T. R. (2006). Extrasynaptic membrane trafficking regulated by GluR1 serine 845 phosphorylation primes AMPA receptors for long-term potentiation. J. Biol. Chem. 281 (2), 752–758. Epub 2005/11/08. 10.1074/jbc.M509677200 16272153

[B37] Osten P. Khatri L. Perez J. L. Köhr G. Giese G. Daly C. (2000). Mutagenesis reveals a role for ABP/GRIP binding to GluR2 in synaptic surface accumulation of the AMPA receptor. Neuron 27 (2), 313–325. Epub 2000/09/14. 10.1016/s0896-6273(00)00039-8 10985351

[B38] Park J. S. Voitenko N. Petralia R. S. Guan X. Xu J. T. Steinberg J. P. (2009). Persistent inflammation induces GluR2 internalization via NMDA receptor-triggered PKC activation in dorsal horn neurons. J. Neurosci. 29 (10), 3206–3219. Epub 2009/03/13. 10.1523/jneurosci.4514-08.2009 19279258 PMC2664544

[B39] Park M. (2018). AMPA receptor trafficking for postsynaptic potentiation. Front. Cell Neurosci. 12, 361. Epub 2018/10/27. 10.3389/fncel.2018.00361 30364291 PMC6193507

[B40] Park P. Sanderson T. M. Amici M. Choi S. L. Bortolotto Z. A. Zhuo M. (2016). Calcium-permeable AMPA receptors mediate the induction of the protein kinase A-dependent component of long-term potentiation in the Hippocampus. J. Neurosci. 36 (2), 622–631. Epub 2016/01/14. 10.1523/jneurosci.3625-15.2016 26758849 PMC4710778

[B41] Patten S. A. Ali D. W. (2009). PKCgamma-induced trafficking of AMPA receptors in embryonic zebrafish depends on NSF and PICK1. Proc. Natl. Acad. Sci. U. S. A. 106 (16), 6796–6801. Epub 2009/04/16. 10.1073/pnas.0811171106 19366675 PMC2672538

[B42] Reiss A. B. Arain H. A. Stecker M. M. Siegart N. M. Kasselman L. J. (2018). Amyloid toxicity in Alzheimer's disease. Rev. Neurosci. 29 (6), 613–627. Epub 2018/02/16. 10.1515/revneuro-2017-0063 29447116

[B43] Sheng M. Kim M. J. (2002). Postsynaptic signaling and plasticity mechanisms. Science, 298. , 776–780. Epub 2002/10/26. 10.1126/science.1075333 12399578

[B44] Shi S. Hayashi Y. Esteban J. A. Malinow R. (2001). Subunit-specific rules governing AMPA receptor trafficking to synapses in hippocampal pyramidal neurons. Cell 105 (3), 331–343. Epub 2001/05/12. 10.1016/s0092-8674(01)00321-x 11348590

[B45] Smriga M. Saito H. Nishiyama N. (1995). Hoelen (Poria Cocos Wolf) and ginseng (Panax Ginseng C. A. Meyer), the ingredients of a Chinese prescription DX-9386, individually promote hippocampal long-term potentiation *in vivo* . Biol. Pharm. Bull. 18 (4), 518–522. Epub 1995/04/01. 10.1248/bpb.18.518 7655419

[B46] Takahashi R. H. Yokotsuka M. Tobiume M. Sato Y. Hasegawa H. Nagao T. (2021). Accumulation of cellular prion protein within β-amyloid oligomer plaques in aged human brains. Brain pathol. (Zurich, Switz. 31 (5), e12941. Epub 2021/02/25. 10.1111/bpa.12941 PMC8412093 33624334

[B47] Tan H. L. Chiu S. L. Zhu Q. Huganir R. L. (2020). GRIP1 regulates synaptic plasticity and learning and memory. Proc. Natl. Acad. Sci. U. S. A. 117 (40), 25085–25091. Epub 2020/09/20. 10.1073/pnas.2014827117 32948689 PMC7547244

[B48] Watson J. F. Ho H. Greger I. H. (2017). Synaptic transmission and plasticity require AMPA receptor anchoring via its N-terminal domain. Elife. 6, e23024. Epub 2017/03/16. 10.7554/eLife.23024 28290985 PMC5370185

[B49] Wilcox K. C. Lacor P. N. Pitt J. Klein W. L. (2011). Aβ oligomer-induced synapse degeneration in Alzheimer's disease. Cell. Mol. Neurobiol. 31 (6), 939–948. Epub 2011/05/04. 10.1007/s10571-011-9691-4 21538118 PMC3146579

[B50] Xu Y. M. Wang X. C. Xu T. T. Li H. Y. Hei S. Y. Luo N. C. (2019). Kai Xin San ameliorates scopolamine-induced cognitive dysfunction. Neural Regen. Res. 14 (5), 794–804. Epub 2019/01/29. 10.4103/1673-5374.249227 30688265 PMC6375048

[B51] Zhang B. Li Y. Liu J. W. Liu X. W. Wen W. Cui Y. (2019). Postsynaptic GluR2 involved in amelioration of Aβ-induced memory dysfunction by Kaixin-San through rescuing hippocampal LTP in mice. Rejuvenation Res. 22 (2), 131–137. Epub 2018/07/17. 10.1089/rej.2018.2080 30009679

[B52] Zhang Y. Saito H. Nishiyama N. Abe K. (1994). Effects of DX-9386, a traditional Chinese medicinal prescription, on long-term potentiation in the dentate gyrus in rats. Biol. Pharm. Bull. 17 (10), 1337–1340. Epub 1994/10/01. 10.1248/bpb.17.1337 7874054

[B53] Zhang Y. Zhu M. Sun Y. Tang B. Zhang G. An P. (2021). Environmental noise degrades hippocampus-related learning and memory. Proc. Natl. Acad. Sci. U. S. A. 118 (1), e2017841117. Epub 2020/11/25. 10.1073/pnas.2017841117 33229555 PMC7797896

