# Supplementary material for: Kaixin-San improves Aβ-induced synaptic plasticity inhibition by affecting the expression of regulation proteins associated with postsynaptic AMPAR expression
Source: Front Pharmacol. 2023 Feb 14;14:1079400. doi: 10.3389/fphar.2023.1079400 (PMC9970989; doi:10.3389/fphar.2023.1079400)

***Supplementary Material***

# Supplementary Figure


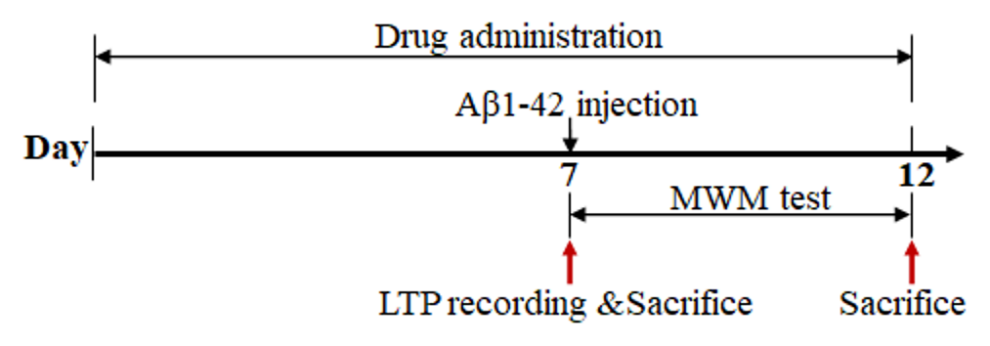


**Supplementary Figure.** The experimental procedure used in this study.

# Graphical Abstract


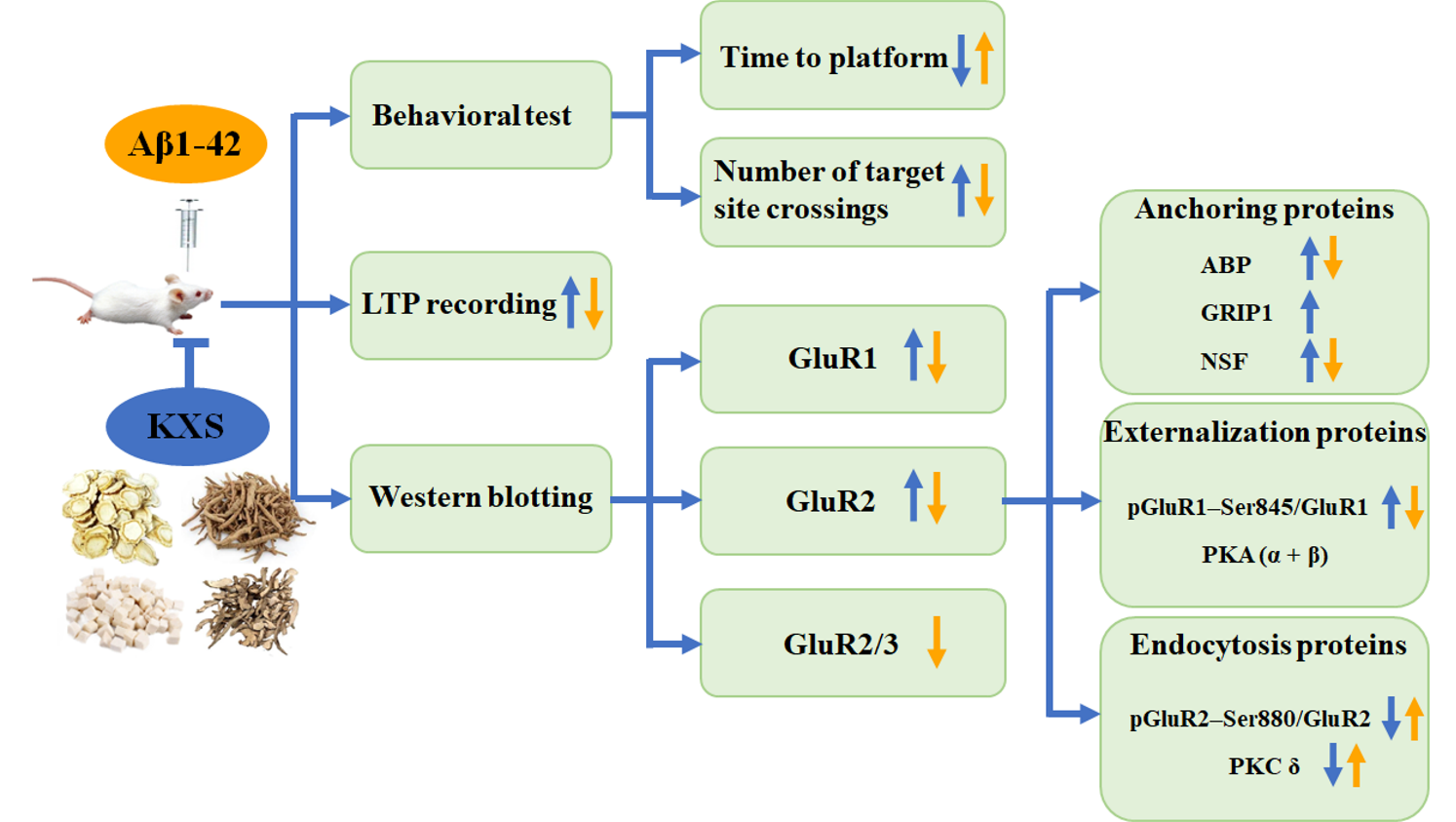

Supplement: Supplementary file 1 [file DataSheet1.docx]
